# Synchronous In Situ Harmonic Calibration of Vibration Amplitude and Gap in 2D Nanomechanical Resonators

**DOI:** 10.3390/nano16171113

**Published:** 2026-09-03

**Authors:** Yuchen Zhang, Ying Liu, Tianyi Zhang, Zhiyu Guo, Yang Xiao, Jun Zhou, Feng Hu, Fang Luo, Shiqiao Qin

**Affiliations:** 1 Hunan Provincial Key Laboratory of Novel Nano-Optoelectronic Information Materials and Devices, College of Advanced Interdisciplinary Studies, National University of Defense Technology, Changsha 410073, China; z_yc98@nudt.edu.cn (Y.Z.); hufeng@nudt.edu.cn (F.H.); 2 Nanhu Laser Laboratory, National University of Defense Technology, Changsha 410073, China; 3 College of Intelligence Science and Technology, National University of Defense Technology, Changsha 410073, China; 4 College of Aerospace Science and Engineering, National University of Defense Technology, Changsha 410073, China

**Keywords:** 2D nanomechanical resonators, nonlinear optical calibration, synchronous multi-harmonic detection, configurational relaxation

## Abstract

Reliable calibration that converts transduced signals into physical displacement is essential for quantitative studies and applications of nanoelectromechanical resonators, including nonlinear dynamics, precision sensing, and optomechanical and electromechanical coupling. Existing harmonic calibration based on nonlinear optical transduction is generally restricted to systems with optically thin suspended layers and highly reflective substrates. Their weak higher-order harmonic signals are also susceptible to noise, drift, and inconsistencies between separately acquired frequency sweeps. Here, we generalize this approach to hexagonal boron nitride/graphene (h-BN/Gra) heterostructure resonators without local metallic reflectors. Multilayer thin-film interference calculations show that a branch-local phase correction enables the effective two-beam inversion to recover vibration amplitude and local static gap within acceptable error bounds. Experimentally, we use multi-demodulator lock-in detection to acquire the ω, 2ω, and 3ω optical responses simultaneously at each frequency point. Ratios among these harmonics then yield frequency-resolved vibration amplitude and local static gap. Repeated frequency sweeps at a constant gate bias simultaneously track the resonance characteristics and local static gap, revealing a time-dependent relaxation of approximately 24 nm in the local device configuration. This work provides a practical in situ route for simultaneously resolving resonance characteristics and configurational evolution across a broader range of nanomechanical resonator architectures.

## 1. Introduction

Nanoelectromechanical resonators, with their ultralow effective masses, high resonance frequencies, and broad frequency tunability [1,2,3], have emerged as versatile mechanical platforms for quantum technologies [4], precision sensing [5,6], and optomechanical and electromechanical coupling [7,8]. Their resonant motion is typically read out through electrical or optical signals, such as voltage or optical intensity, whose relationship to displacement can become nonlinear [2,9]. More detailed quantitative studies of resonator dynamics require these transduced signals to be calibrated into physical displacement, particularly as the response evolves from linear to nonlinear motion [10,11,12]. For example, the vibration amplitude reflects the resonator’s operating regime, whereas the local static gap characterizes the device configuration under operation or external control [13]. Reliable calibration therefore provides the essential link between measured transduction signals and the actual mechanical behavior of suspended nanoelectromechanical resonators and, more broadly, constitutes a prerequisite for high-precision dynamical analysis and device performance optimization.

Several calibration strategies have been developed for nanomechanical resonators. Thermomechanical calibration converts a measured noise spectrum into displacement through the equipartition theorem, but requires accurate values for the resonator temperature and effective mode mass, which can be difficult to establish for two-dimensional membranes [14]. Other approaches based on model fitting or reference optical measurements can also provide signal-to-displacement conversion, but they usually require dedicated reference measurements or independently constrained optical models [15,16]. In conventional small-signal measurements, the conversion factor is often determined from the local linear optical responsivity near the operating point. As the vibration amplitude increases or the operating point moves beyond this locally linear region, nonlinear optical transduction can introduce systematic bias into the displacement estimate [17]. Harmonic calibration based on nonlinear optical transduction is particularly attractive because, in its ideal two-beam form, it requires only the wavelength of the detection laser and no additional prior knowledge of the resonator’s mechanical properties, actuation force, geometric distances, or laser intensity [9]. Because the laser wavelength is readily known in routine experiments, this requirement is easily satisfied, enabling in situ extraction of vibration amplitude and local static gap during resonance measurements. However, extending this approach to measurements in more general nanoelectromechanical systems (NEMS) devices requires addressing two issues: whether analytical inversion remains valid beyond the optically thin-suspended-layer and high-reflectivity-substrate approximations, and how weak higher-harmonic signals can be extracted accurately when noise, drift, and inconsistencies across separately acquired frequency sweeps introduce additional uncertainty.

In this work, by combining multilayer thin-film interference modeling and multi-demodulator synchronous lock-in detection, we extend nonlinear optical harmonic calibration to more general nanoelectromechanical resonator structures that do not use a local metallic gate as a high-reflectivity mirror. Within explicitly defined calibration windows, a branch-local phase correction allows a convenient effective two-beam model to recover the vibration amplitude and local static gap within an acceptable error range. Experimentally, multiple harmonic responses with consistent resonance line shapes and phase relations are extracted at each excitation-frequency point during the fundamental-mode frequency sweep, and the calibrated vibration amplitude and local static gap show frequency-window self-consistency. Finally, applying the method to repeated resonance sweeps under a constant gate bias enables synchronous tracking of the observed resonance peak frequency and local static gap, revealing an approximately 24 nm relaxation of the local device configuration over time. Our work provides a straightforward, in situ approach for synchronously resolving resonance characteristics and device-configuration evolution in a broader range of 2D nanoelectromechanical resonator structures.

## 2. Methods

### 2.1. Device Fabrication

**Figure 1:**
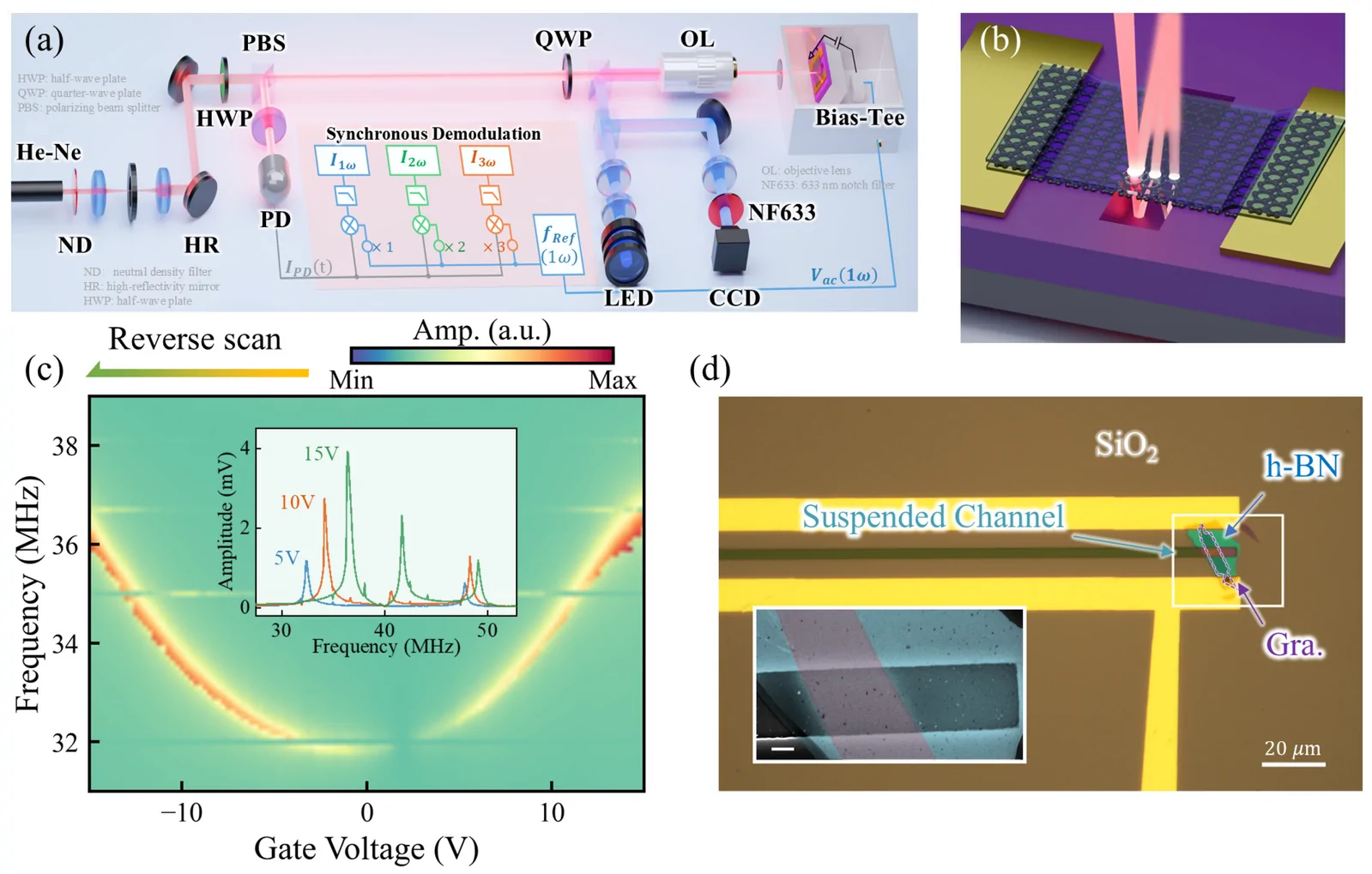
Synchronous multi-harmonic detection scheme and gate-voltage-dependent frequency dispersion of the h-BN/Gra resonator. (**a**) Electro-optical resonance measurement setup for synchronous detection of the fundamental and higher-harmonic responses. (**b**) Schematic of the h-BN/Gra heterostructure resonator suspended over an etched SiO_2_ trench and the corresponding multilayer thin-film interference geometry. (**c**) Two-dimensional color plot of the frequency dispersion of the resonator. The color scale represents the modulation amplitude of the reflected laser intensity induced by membrane motion. Inset: representative frequency-sweep traces at selected gate voltages. (**d**) Top-view optical micrograph of the h-BN/Gra resonator. Inset: false-color SEM image highlighting the suspended region (scale bar: 1 μm). Blue indicates h-BN and purple indicates graphene.

The device platform was fabricated from a heavily doped silicon (Si) wafer capped with a 300 nm thermal silicon dioxide (SiO_2_) layer. Cr/Au contact electrodes (5/50 nm) were first defined by electron-beam lithography and deposited on the oxide surface by electron-beam evaporation. A second electron-beam lithography step defined a rectangular etching window in the oxide region between the electrodes, followed by inductively coupled plasma (ICP) dry etching to form an etched trench with a width of 3 μm and a depth of 260 nm. The h-BN/Gra heterostructure was then assembled and released onto the trench region using our previously reported modified polydimethylsiloxane (PDMS)-assisted polycarbonate (PC) pick-up transfer process [18,19], completing the heterostructure stacking and resonator assembly within the same transfer workflow. Optical microscopy and SEM imaging confirmed the spatial layout of the heterostructure membrane and the well-defined suspended region (Figure 1d and inset), with h-BN and graphene distinguished in the false-colored SEM image as blue and purple regions, respectively.

### 2.2. Measurement Setup

The mechanical response of the suspended heterostructure resonator was measured using the electro-optical setup shown in Figure 1a at room temperature and under a vacuum pressure of approximately $5\times 10^{-3}\;\mathrm{mbar}$. A DC gate voltage ($V_{g}^{DC}$) combined with a radio-frequency excitation ($V_{drive}^{AC}\left(1\omega \right)$) was applied to the doped-Si back gate. In the capacitor formed by the suspended h-BN/Gra membrane and the underlying back gate, the DC component generated an electric field that exerted a downward electrostatic force on the membrane, causing it to deflect and become tensioned. The MHz-frequency RF excitation supplied by an HF2LI lock-in amplifier (Zurich Instruments) periodically modulated the electrostatic force acting on the tensioned membrane, thereby driving the resonator into resonance.

The membrane motion was read out by monitoring the dynamic reflectance of a focused λ=632.8 nm He-Ne laser spot on the suspended h-BN/Gra heterostructure with a fast photodetector [18,20,21]. Because the reflected intensity is a nonlinear function of membrane displacement, the multiple demodulators of the lock-in amplifier enabled synchronous extraction of the fundamental and higher-harmonic optical responses at each excitation frequency. Figure 1b shows a schematic diagram of the hexagonal boron nitride/graphene (h-BN/Gra) heterostructure resonator we designed. Further details of the device fabrication and measurement setup are provided in Section S.I of the Supplementary Information.

Figure 1c shows a two-dimensional color plot of the gate-voltage-dependent frequency dispersion of the resonator. The color scale represents the modulation amplitude of the reflected laser intensity associated with membrane vibration amplitude. The resonance modes exhibit a clear and smooth positive frequency dispersion as the gate voltage is varied, indicating a relatively low built-in tensile stress in the suspended membrane [22,23]. This behavior is also consistent with a well-suspended and mechanically uniform h-BN/Gra membrane, as jointly supported by the smooth modal dispersion and the false-colored SEM image inset in Figure 1d. Representative frequency-sweep traces at selected gate voltages, shown in the inset of Figure 1c, reveal consistent positive dispersion of several resonance modes over a wider frequency range and further confirm that the main mode discussed in this work, near 34 MHz, can be clearly resolved and tracked within the measurement window.

### 2.3. Effective Two-Beam Harmonic Calibration

Assuming that the suspended membrane behaves as an optically thin layer and that the back reflector is ideal, the reflected optical intensity used to transduce the motion of the NEMS resonator can be expressed as follows (Equation (1)) [9]:

(1) $$I\left(t\right)=A+B\cos\left[\gamma \left(g+\overset{ˇ}{x}\left(t\right)\right)\right]$$

where γ=4π/λ, denoting the optical wavelength; g denotes the local static gap between the membrane and the back reflector; and $\overset{ˇ}{x}\left(t\right)$ represents the time-dependent membrane displacement associated with its resonant motion.

For the single-frequency motion of a suspended two-dimensional material membrane,

(2) $$\overset{ˇ}{x}\left(t\right)=\delta \sin\left(\omega t\right)$$

defining z ≜ γδ, where δ is the oscillation amplitude, the reflected-intensity signal can be expanded using the Jacobi–Anger identity:

(3) $$\begin{array}{l} I\left(t\right)=\\ A+B\cos\left(\gamma g\right)J_{0}\left(z\right)\\ -2B\sin\left(\gamma g\right)J_{1}\left(z\right)\sin\left(\omega t\right)\\ +2B\cos\left(\gamma g\right)J_{2}\left(z\right)\cos\left(2\omega t\right)\\ -2B\sin\left(\gamma g\right)J_{3}\left(z\right)\sin\left(3\omega t\right)+\ldots \end{array}$$

The first three harmonic components at ω, 2ω, and 3ω, where ω is the drive frequency, can therefore be expressed as follows:

(4) $$I_{1\omega }=-2B\sin\left(\gamma g\right)J_{1}\left(z\right)$$

(5) $$I_{2\omega }=2B\cos\left(\gamma g\right)J_{2}\left(z\right)$$

(6) $$I_{3\omega }=-2B\sin\left(\gamma g\right)J_{3}\left(z\right)$$

The corresponding harmonic-amplitude ratios are:

(7) $$\frac{I_{3\omega }}{I_{1\omega }}=\frac{J_{3}\left(z\right)}{J_{1}\left(z\right)}$$

(8) $$\frac{I_{2\omega }}{I_{1\omega }}=-\cot\left(\gamma g\right)\frac{J_{2}\left(z\right)}{J_{1}\left(z\right)}\Rightarrow tan\gamma g=\frac{J_{2}\left(z\right)}{J_{1}\left(z\right)}\cdot \frac{I_{1\omega }}{I_{2\omega }}$$

The third-to-first harmonic ratio depends only on the oscillation amplitude δ through z= γδ. Once δ has been determined from this ratio, the static gap g can be obtained by additionally using the second-to-first harmonic ratio. With the small-argument approximations $J_{1}≃z/2-z^{3}/16$, $J_{2}≃z^{2}/8-z^{4}/96$, and $J_{3}≃z^{3}/48$, simplified expressions for calibrating the oscillation amplitude and static gap can be derived within the effective two-beam interference model:

(9) $$\delta =\frac{2\sqrt{6}}{\gamma }\sqrt{\frac{I_{3\omega }/I_{1\omega }}{1+3I_{3\omega }/I_{1\omega }}}$$

(10) geff=1γnπ+tan−112γδ−γ3δ36γ2δ2−48I2ωI1ω,n∈Z

It is worth noting that, in the experiments, the lock-in amplifier reports the magnitudes of the harmonic signals rather than the signed Fourier coefficients appearing in the analytical expressions. The loss of sign information introduces a sign, or equivalently branch, ambiguity when the effective static gap $g_{\mathrm{eff}}$ is extracted from the second-to-first harmonic ratio. A detailed discussion of this ambiguity is provided in Section S.II of the Supplementary Information.

Accordingly, defining $L≜\gamma^{-1}\pi \;=\;\lambda /4\;=\;158.2\;nm$, the final expression used to calibrate the local effective static gap within the effective two-beam interference model is

(11) $$g_{\mathrm{eff}}=nL+\left\{\begin{array}{ll} \frac{1}{\gamma }\tan^{-1}\left(\frac{12\gamma \delta -\gamma^{3}\delta^{3}}{\left(6\gamma^{2}\delta^{2}-48\right)r_{21}}\right) & ,\;\;g_{\mathrm{eff}}\in \left[nL-\frac{L}{2}\right.,\left.nL\right)\\ \frac{1}{\gamma }\tan^{-1}\left(\frac{12\gamma \delta -\gamma^{3}\delta^{3}}{\left(6\gamma^{2}\delta^{2}-48\right)\left(-r_{21}\right)}\right) & ,\;\;g_{\mathrm{eff}}\in \left(nL\right.,\left.nL+\frac{L}{2}\right) \end{array}\right.,n\in Z$$

In addition, Section S.III of the Supplementary Information presents a worked example demonstrating how Equations (9) and (11) are used to calibrate the oscillation amplitude and static gap from the experimentally measured harmonic-amplitude ratios.

### 2.4. Multilayer Thin-Film Interference Model

Although Equations (9) and (11), derived from the effective two-beam interference model, provide a straightforward harmonic-based calibration method that requires no additional a priori information, this simplified model may not accurately describe more general two-dimensional (2D) nanomechanical resonators, including the device investigated here. Such devices may lack a highly reflective metallic back reflector beneath the suspended 2D material. Moreover, real suspended 2D materials and heterostructures have finite thicknesses and complex refractive indices and may exhibit optical absorption and reflections at the interfaces between constituent layers.

For such multi-interface interference structures, a multilayer thin-film interference model provides a more accurate framework for calculating the amplitude reflection coefficient and optical intensity reflectance [17,24,25]. This model uses a recursive procedure to calculate, layer by layer, the effective amplitude reflection coefficient of the underlying composite stack as viewed from the adjacent upper layer. Taking our h-BN/Gra heterostructure resonator as an example, the device comprises six layers along the direction of light incidence: vacuum, h-BN, Gra, vacuum, SiO_2_, and Si. The corresponding structural parameters, complex refractive indices, and their sources are provided in Table S1.

The amplitude reflection coefficient at the interface between media j and j+1 is given by the Fresnel equation at normal incidence:

(12) $$r_{j,j+1}=\frac{N_{j}-N_{j+1}}{N_{j}+N_{j+1}}$$

For the bottom SiO_2_/Si stack, its effective amplitude reflection coefficient as viewed from the fourth layer (vacuum) can be obtained by combining the reflection coefficients $r_{4,5}$ and $r_{5,6}$ with the thin-film interference occurring in the fifth layer:

(13) $${r_{4,5}}^{\mathrm{eff}}=\frac{r_{4,5}+r_{5,6}exp\left(2i\beta_{5}\right)}{1+r_{4,5}r_{5,6}exp\left(2i\beta_{5}\right)}$$

Here, the phase thickness $\beta_{5}$ is determined by the thickness $d_{5}$ of the fifth layer and is given by $\beta_{5}=\frac{2\pi }{\lambda }{\tilde{n}}_{5}d_{5}$.

The resulting effective amplitude reflection coefficient of the SiO_2_/Si stack is then substituted into the thin-film interference calculation for the overlying fourth layer (vacuum). This yields the effective reflection coefficient of the combined vacuum/SiO_2_/Si stack as viewed from the next upper layer, Gra. Repeating this recursive procedure for each successive layer ultimately gives the effective amplitude reflection coefficient ${r_{1,2}}^{\mathrm{eff}}$ of the complete multilayer structure. The optical intensity reflectance of the resonator for the specified structural and optical parameters can then be expressed as

(14) $$R_{TF}={\left|{r_{1,2}}^{\mathrm{eff}}\right|}^{2}$$

### 2.5. Branch-Local Phase Correction

To generalize the experimentally convenient harmonic calibration based on the effective two-beam model to more general structures, such as the h-BN/Gra resonator studied here, we introduce a branch-dependent local phase correction.

Calculations of the effective amplitude reflection coefficient of silicon substrates covered with SiO_2_ layers of different thicknesses, viewed from the vacuum side (see Section S.V of the Supplementary Information), show that a key difference from an ideal local metallic back reflector with high reflectivity is that the amplitude reflection coefficient of the SiO_2_/Si structure is complex. Consequently, the reflected optical field acquires an additional phase shift. To account for this effect, we introduce a phase correction φ into the ideal effective two-beam model:

(15) $$R\left(g\right)=A+Bcos\left(\gamma g+\varphi \right)$$

Furthermore, real suspended 2D materials and heterostructures have finite thicknesses and complex refractive indices and may exhibit optical absorption and reflections at the interfaces between constituent layers. These effects break the symmetry between the incident and reflected optical fields assumed in the ideal two-beam interference model. We therefore generalize the phase correction to a branch-dependent local quantity $\varphi_{n}$:

(16) $$R_{n}\left(g\right)=A_{n}+B_{n}cos\left(\gamma g+\varphi_{n}\right)$$

The physical local static gap g can then be recovered by inverting the ideal model with the branch-dependent local phase correction, which gives

(17) $$g=g_{eff}-\frac{\varphi_{n}}{\gamma }$$

## 3. Results

**Figure 2:**
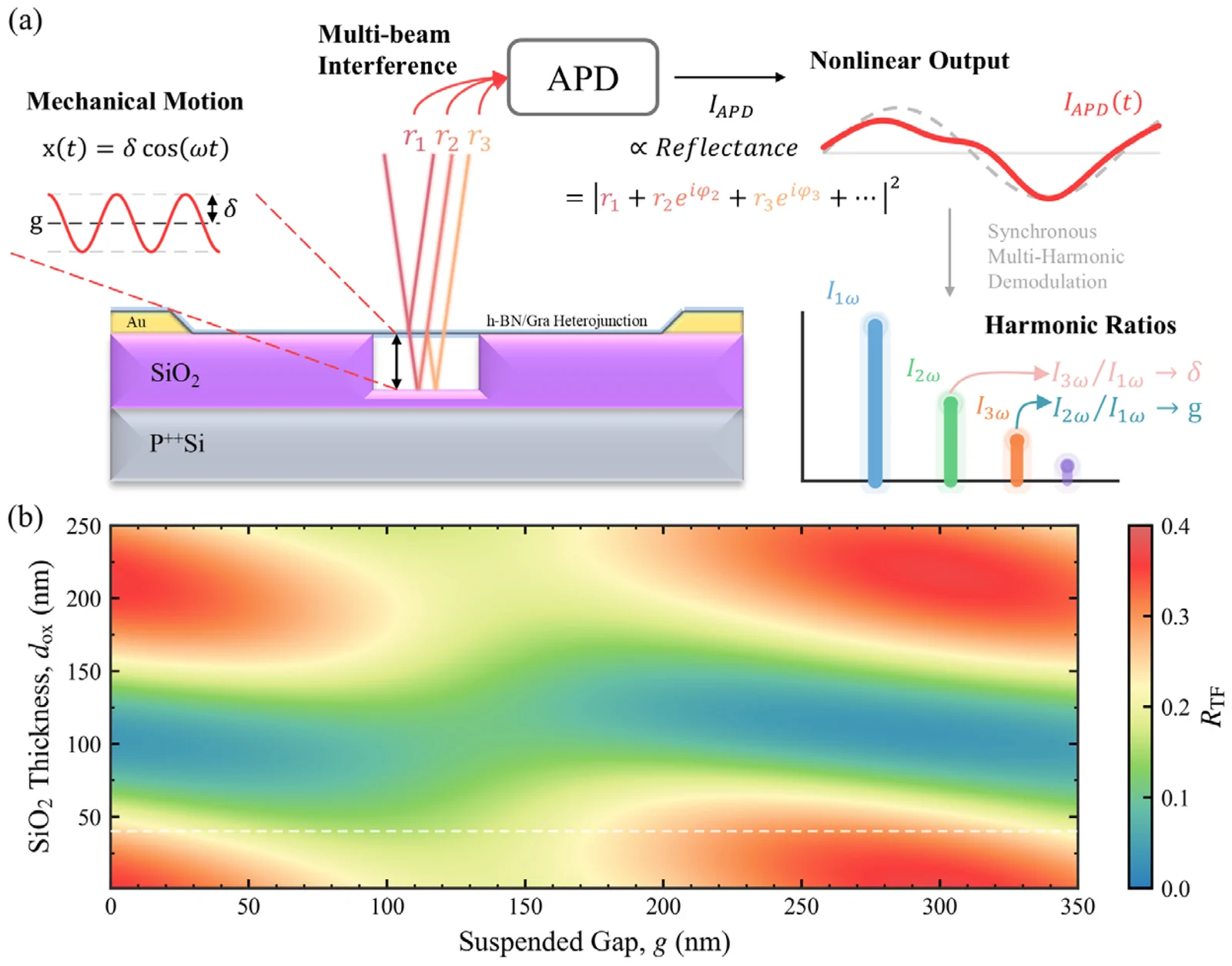
Nonlinear optical transduction and reflectance landscape from a multilayer thin-film interference model for optical harmonic calibration. (**a**) Schematic of nonlinear optical transduction from single-tone mechanical motion to synchronous higher-harmonic photodetector responses, with harmonic ratios used to calibrate the vibration amplitude and local static gap (Equations (9) and (11)). (**b**) Reflectance landscape calculated with the multilayer thin-film interference model as a function of the local static gap g and SiO_2_ thickness $d_{ox}$. Dashed guides mark the device-relevant $d_{ox}=40\;\mathrm{nm}$ slice used for subsequent harmonic calibration analysis.

**Figure 3:**
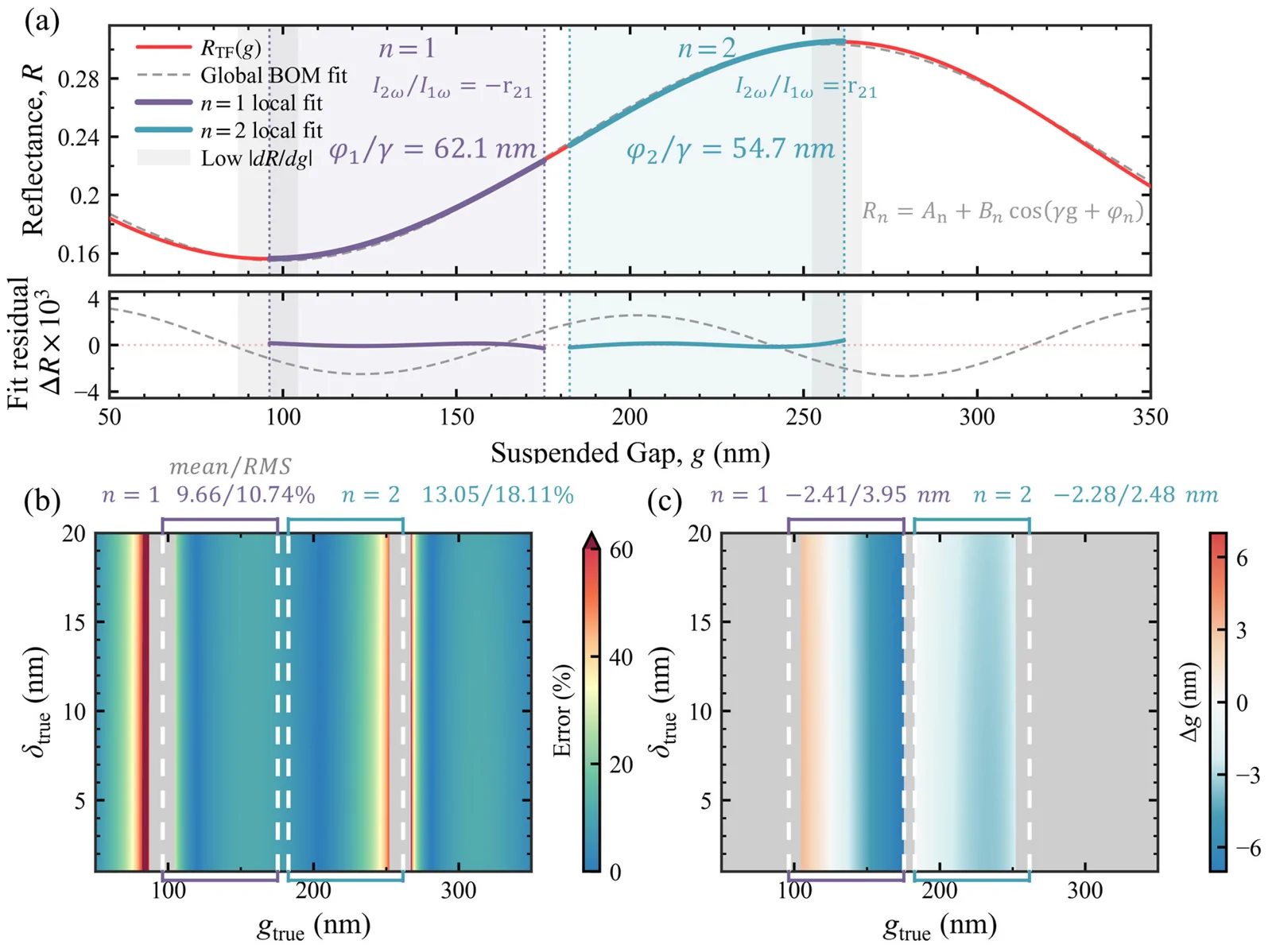
Validation of effective two-beam inversion for amplitude and gap calibration in realistic device structures using a multilayer thin-film interference model. (**a**) Multilayer thin-film reflectance fitted by global and branch-local effective two-beam models for the $n=1,-r_{21}$ and $n=2,+r_{21}$ calibration branches; residuals are shown below. (**b**,**c**) Relative error map of vibration-amplitude calibration, $\left|∆\delta \right|/\delta_{true}$ (**b**), and error map of local static-gap calibration, $∆g=g_{recovered}-g_{true}$ (**c**), obtained by applying branch-local effective two-beam inversion to harmonic responses generated from the multilayer thin-film interference model.

Optical harmonic calibration based on nonlinear transduction relies on a separation between mechanical motion and optical readout. A membrane that moves predominantly at a single mechanical frequency, $x\left(t\right)=\delta cos\left(\omega t\right)$, can still generate photodetector components at 2ω,3ω,4ω and higher orders if the reflected intensity is a nonlinear function of the local gap, $R\left[g+x\left(t\right)\right]$. Higher-harmonic photodetector signals are therefore not, by themselves, evidence of substantial mechanical motion at higher harmonics. Furthermore, the effects of parasitic mechanical nonlinearities that may be introduced by capacitive actuation [26], as well as internal resonances between eigenmodes [11,27,28], must be considered carefully [29,30]. For the electrical actuation scheme used here, as described in Section 2.2, the electrostatic force scales as $F_{es}\propto {\left[V_{g}^{DC}+V_{drive}^{AC}cos\left(\omega t\right)\right]}^{2}$, and it contains a force component at 2ω that can directly drive a mechanical mode when the excitation frequency approaches half of the mode’s resonance frequency. Control measurements over a broad frequency range confirmed the presence of this electrostatic 2ω excitation pathway. However, no resonantly enhanced parasitic mechanical pathway was observed near integer multiples of the resonance frequency of the primary mode studied here. Under this condition, the measured higher-harmonic optical signals mainly encode local derivatives of the optical transfer function and can be used as calibration observables for extracting δ and g (using Equations (9) and (11)), as illustrated in Figure 2a. To place this nonlinear transduction in an optical environment relevant to the actual device, we calculated the reflectance landscape using a multilayer thin-film interference model [15,24,25] (Figure 2b; see Section S.IV of the Supplementary Information for parameter details). The multilayer thin-film model treats the reflected intensity as a function of the local static gap g and the residual SiO_2_ thickness $d_{ox}$, which we denote as $R_{TF}\left(g,d_{ox}\right)$. Here, $d_{ox}$ specifically denotes the SiO_2_ thickness between the suspended h-BN/Gra heterostructure and the heavily doped silicon gate, excluding the vacuum gap. The $d_{ox}=40\;\mathrm{nm}$ slice is the one-dimensional cross-section of this reflectance landscape at fixed $d_{ox}=40\;\mathrm{nm}$, corresponding to the actual residual SiO_2_ thickness at the trench bottom of our device. As indicated by the dashed line in Figure 2b, this cross-section is replotted in Figure 3a. It shows that the reflected intensity retains a pronounced dependence on the local gap coordinate even in the absence of an ideal high-reflectivity back mirror. This result raises the central quantitative question for the following analysis: whether the analytically convenient effective two-beam inversion formula remains usable in multilayer thin-film structures representative of common device geometries such as ours.

Since the device reflectance is determined simultaneously by interference at multiple optical interfaces, the optically thin-suspended-layer and highly reflective-substrate approximations underlying the ideal two-beam calibration model are not strictly satisfied. To assess whether the analytical inversion based on the effective two-beam model remains applicable beyond this ideal limit, we used the multilayer thin-film interference model as a forward model to calculate $R_{TF}\left(g\right)$ and the harmonic responses generated for prescribed values of $g_{true}$ and $\delta_{true}$, and then compared the resulting reflectance with global and branch-local effective two-beam fits (Figure 3a). Within each calibration branch, the multilayer reflectance was approximated as $R_{TF}\left(g\right)\approx A_{n}+B_{n}cos\left(\gamma g+\varphi_{n}\right)$, where $\gamma =\frac{4\pi }{\lambda }$; $A_{n}$, $B_{n}$ and $\varphi_{n}$ account for the mean reflectance, modulation depth and effective optical phase offset, respectively. The fitted phase $\varphi_{n}$ was subsequently used to map the analytical branch coordinate onto the physical gap coordinate according to $g=g_{eff}-\frac{\varphi_{n}}{\gamma }$. The residual curves in the lower panel of Figure 3a show that branch-local models incorporating local phase corrections accurately reproduced the multilayer reflectance within their respective calibration windows, with $R^{2}>0.99996$ and maximum absolute reflectance residuals below $4\times 10^{-4}$, while substantially suppressing the systematic deviations retained by the global fit.

Having established the accuracy of the branch-local approximation, we next quantified how the remaining model mismatch propagated into the recovered vibration amplitude and local static gap. Using the same multilayer forward model at λ=632.8 nm and $d_{ox}=40\;\mathrm{nm}$, we generated harmonic responses over $g_{true}=50-350\;\mathrm{nm}$ and $\delta_{true}=1-20\;\mathrm{nm}$ and subsequently recovered δ and g using effective two-beam inversion with branch-local phase correction. Because the inversion becomes intrinsically ill-conditioned as the optical responsivity approaches zero, regions satisfying $\left|\frac{\partial R_{TF}}{\partial g}\right|\leq 15\%max\left(\left|\frac{\partial R_{TF}}{\partial g}\right|\right)$ were excluded from the main statistics. Figure 3b,c show the resulting absolute relative amplitude error, $\frac{\left|\Delta \delta \right|}{\delta_{true}}$, and local static-gap error, $\Delta g=g_{recovered}-g_{true}$, respectively. Within the $n=1,-r_{21}$ calibration window, the mean and RMS absolute relative amplitude errors were 9.66% and 10.74%, respectively, whereas the mean signed and RMS gap errors were −2.41 nm and 3.95 nm. The corresponding values for the $n=2,+r_{21}$ branch were 13.05% and 18.11% for the amplitude calibration and −2.28 nm and 2.48 nm for the gap calibration.

**Figure 4:**
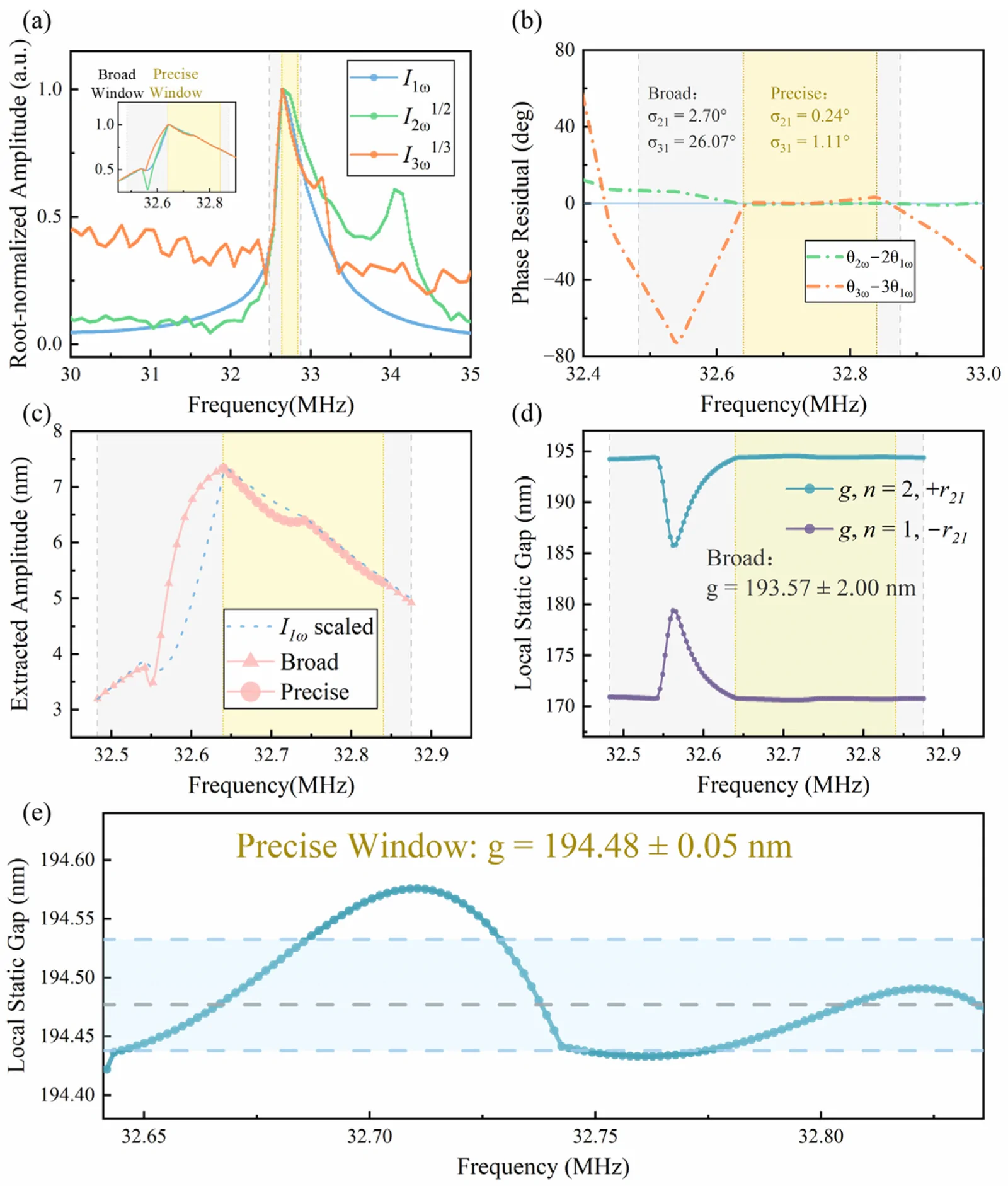
Synchronous multi-harmonic amplitude and gap calibration with frequency-window self-consistency. (**a**) Root-normalized line shapes of the synchronously measured 1ω, 2ω, and 3ω optical responses, showing their common resonance profile near the fundamental resonant mode. (**b**) Phase consistency of the higher-harmonic responses, quantified by the residual phase combinations $\varphi_{2\omega }\;-\;2\varphi_{1\omega }$ and $\varphi_{3\omega }\;-\;3\varphi_{1\omega }$ within the broad and precision frequency windows. (**c**) Frequency-resolved vibration amplitude $\delta \left(f\right)$ calibrated from the synchronous harmonic ratios in the broad and precision windows, with a scaled 1ω response shown for line shape comparison. (**d**) Frequency-resolved local static gap $g\left(f\right)$ calibrated from the synchronous harmonic ratios, showing the candidate calibration branches in the broad window. The trench geometry and continuous gap evolution observed in the subsequent time-relaxation measurements identify $n=2,+r_{21}$ as the physically relevant branch. (**e**) Stability of the selected local static-gap branch in the precision window, with the phase-corrected g= 194.48 ± 0.05 nm shown as mean ± s.d.

**Figure 5:**
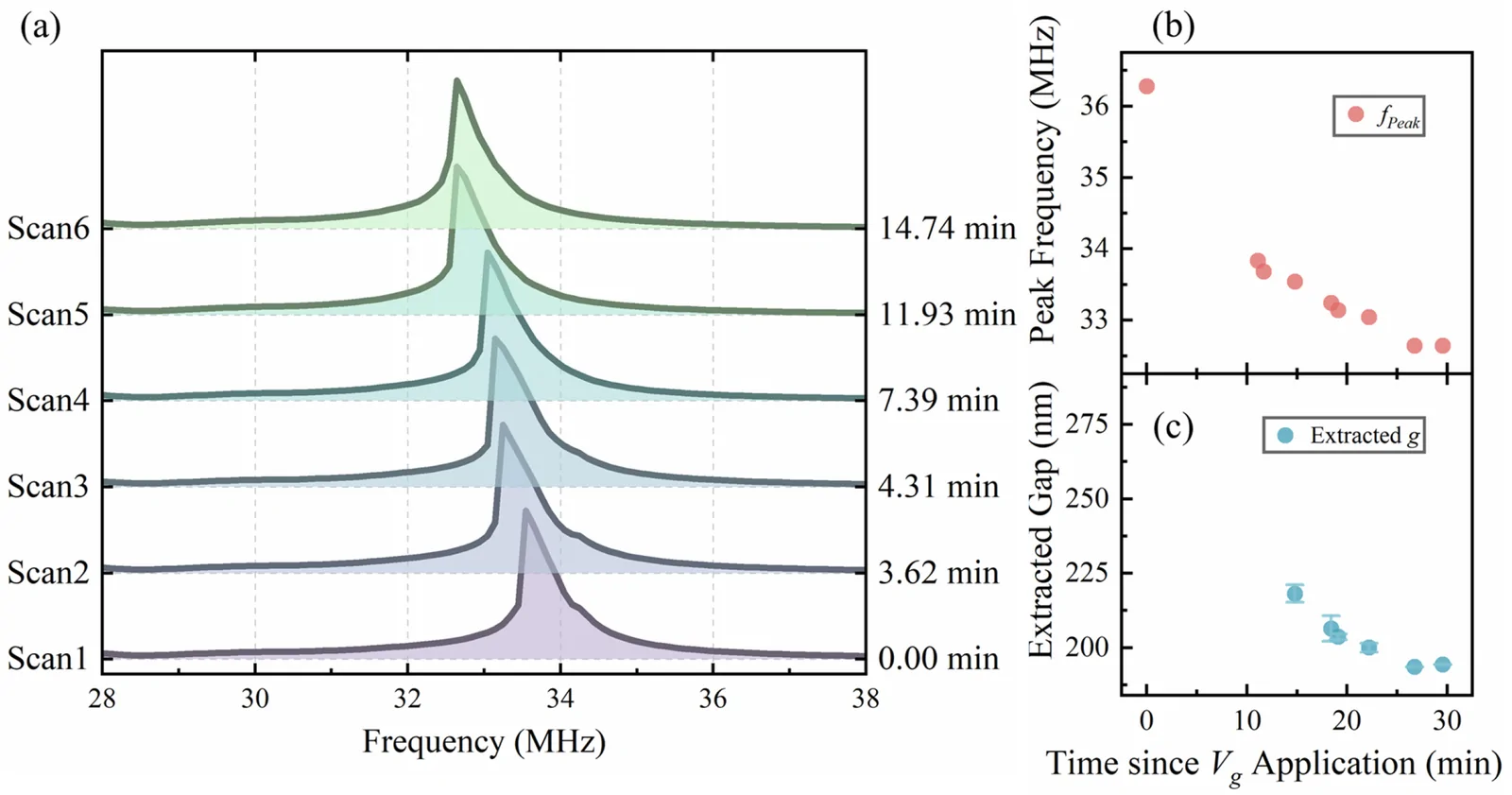
Time-dependent relaxation of resonance peak frequency and local static gap under a constant gate bias captured by synchronous multi-harmonic calibration. (**a**) Six repeated frequency down-sweeps acquired with fixed rf settings under a constant gate bias, shown as a filled ridgeline plot to visualize the relaxation of the resonance peak. (**b**,**c**) Correlated evolution of the observed resonance peak frequency $f_{peak}$ (**b**) and calibrated local static gap g (**c**) over time after applying the constant gate bias of $V_{g}\;=\;15\;V$.

The agreement in Figure 3 should be interpreted as a controlled approximation rather than implying that a real multilayer device can be optically treated as an ideal two-beam interference system. These results show that effective two-beam inversion with branch-local phase correction can recover the vibration amplitude and local static gap within an acceptable range of systematic error. Specifically, a relative amplitude error of approximately 15% corresponds to an absolute error on the order of 1 nm for the several-to −10 nm vibration amplitudes extracted in Figure 4, whereas the gap errors of approximately 2.5–4.0 nm remain substantially smaller than the approximately 25 nm local static-gap evolution observed in Figure 5. Moreover, within each calibration branch, the gap error varies continuously and smoothly with $g_{true}$ and $\delta_{true}$, rather than appearing as point-to-point random fluctuations. The error therefore acts primarily as a smoothly varying systematic bias in the absolute gap, limiting its absolute accuracy without comparably obscuring the resolvability or overall temporal trend of relative gap changes. The error maps also establish the operating boundaries of this approximation: as the optical responsivity decreases or the operating point falls outside the valid calibration window, small uncertainties in the harmonic ratios can be amplified into larger calibration errors. Because the effective phase correction and residual non-cosine distortion depend on the specific optical stack, the corresponding correction parameters and valid calibration windows should be determined from the multilayer thin-film model for each actual device structure. Within these boundaries, calibration based on nonlinear optical transduction retains the analytical convenience of effective two-beam calibration without requiring the device to strictly satisfy the ideal thin-layer and high-reflectivity-substrate assumptions.

We subsequently tested the calibration using experimental harmonic responses acquired synchronously during a frequency down-sweep at $V_{g}=15\;V$ and an rf excitation of $V_{ac}=100\;\mathrm{mV}$ (Figure 4). After taking the first power, square root and cube root of the 1ω, 2ω and 3ω response magnitudes, respectively, and normalizing each trace, the resulting curves exhibited a common resonance line shape near the fundamental mechanical mode (Figure 4a). The residual phase combinations $\varphi_{2\omega }-2\varphi_{1\omega }$ and $\varphi_{3\omega }-3\varphi_{1\omega }$ remained comparatively stable within the selected broad (32.4829–32.8749 MHz) and precision (32.64–32.84 MHz) frequency windows (Figure 4b). Their standard deviations were approximately 2.70° and 26.07° in the broad window and decreased to approximately 0.24° and 1.11° in the precision window, respectively. The common resonance line shapes and increasingly stable phase relations jointly support that the higher-harmonic optical signals used for calibration remain synchronously associated with the same fundamental mechanical response through a common nonlinear optical transduction process.

After complex linear-background subtraction and first-order line shape template fitting, the synchronous harmonic ratios were used to recover the frequency-resolved vibration amplitude $\delta \left(f\right)$ and local static gap $g\left(f\right)$. In both the broad and precision windows, the calibrated $\delta \left(f\right)$ exhibited a resonance line shape similar to the scaled 1ω response shown in Figure 4c. By contrast, the local static gap remained comparatively stable across the frequency window surrounding the resonance. For the selected $n=2,+r_{21}$ branch, branch-local phase correction yielded g=193.57±2.00 nm in the broad window (mean ± s.d.; Figure 4d). The fluctuation on the low-frequency side of the broad window is consistent with the instability jump of a softening Duffing resonator during the frequency down-sweep [12,26]. After excluding this unstable region, the phase-corrected local static gap became nearly frequency independent in the precision window, giving g=194.48±0.05 nm (mean ± s.d.; Figure 4e), consistent with the physical picture of static electrostatic deflection and tensioning under a fixed gate bias combined with dynamic rf excitation. Together, the resonant $\delta \left(f\right)$ and precision-window-stable $g\left(f\right)$ provide a frequency-window self-consistency check of synchronous multi-harmonic amplitude and gap calibration without requiring an external displacement reference.

We next used repeated frequency down-sweeps under a constant gate bias of $V_{g}=15\;V$ to demonstrate in situ local-gap extraction synchronized with resonance-response characterization (Figure 5). Nine down-sweeps were recorded after the gate bias was set. All nine provided a usable fundamental response for tracking the observed resonance peak frequency, whereas the first three scans were acquired while the rf parameters were being optimized and did not provide sufficiently clear higher-harmonic responses for reliable gap inversion. Figure 5a therefore shows the subsequent six sweeps acquired with fixed rf settings. These traces correspond to the final six peak-frequency points in Figure 5b and the six valid phase-corrected local-gap values in Figure 5c.

Quantitative analysis was restricted to these final six scans acquired with fixed rf settings. Over approximately 14.7 min, $f_{peak}:33.545\;\mathrm{MHz}\to 32.642\;\mathrm{MHz}$, corresponding to $\Delta f_{peak}=-0.903\mathrm{MHz}$ or approximately −2.7%; the final two scans reached the same peak position within the frequency resolution of the measurement. During the same sequence, the phase-corrected local static gap followed g:218.2 nm→193.6 nm→194.4 nm: it decreased from 218.2 nm in the first valid calibration scan to 193.6 nm in the fifth scan and remained near 194.4 nm in the sixth scan. The maximum observed decrease was $\Delta g_{max}=-24.6\mathrm{nm}$, whereas the net change between the first and final valid scans was $\Delta g_{final}=-23.8\mathrm{nm}$. This gap evolution is substantially larger than both the 0.05 nm within-window standard deviation obtained in the precision calibration window and the approximately 2.5–4.0 nm model-derived systematic gap error. The correlated decreases in $f_{peak}$ and g therefore indicate a resolvable change in the local configuration of the suspended resonator.

The correlated evolution is consistent with slow configuration relaxation under a constant electrostatic load. Interfacial rearrangement [31], adhesion-mediated relaxation [32], local delamination or slip-like relaxation near the supported region [33,34] could change the effective suspended length, allow the membrane to relax towards a smaller local gap, reduce its in-plane tensile strain and thereby lower the observed resonance peak frequency [26,35,36]. However, the optical calibration measures the local static gap at the laser probe position rather than directly imaging motion at the supported interface. Identifying the microscopic mechanism will therefore require additional control experiments and more comprehensive comparative analyses. Nevertheless, this experiment shows that synchronous multi-harmonic calibration can convert repeated resonance sweeps into a time-resolved, in situ observable of the local device configuration. To our knowledge, this is the first demonstration in a 2D NEMS resonator of in situ tracking of a time-evolving quasi-static local gap synchronized with resonance-response characterization.

## 4. Conclusions

In summary, we extended harmonic calibration based on nonlinear optical transduction to suspended h-BN/Gra heterostructure resonators with realistic multilayer optical structures and without a local metallic gate serving as a high-reflectivity mirror. Multilayer thin-film interference modeling determined the validity window of branch-local effective two-beam inversion and quantified the corresponding amplitude and gap errors. In the experiment, multi-demodulator synchronous lock-in detection provided higher-harmonic optical signals at every frequency point during a frequency sweep, enabling frequency-resolved extraction of the vibration amplitude and local static gap. The *δ*(*f*)-shaped resonant line and the stable phase-corrected *g*(*f*) in the precision window establish a self-consistency check that does not require an external displacement reference. Further, repeated tests under a constant gate bias revealed a correlated relaxation of the observed resonance peak frequency and local static gap, corresponding to an approximately 24 nm local configuration change. Although the detailed mechanism underlying this relaxation remains to be established, our work provides a straightforward, in situ approach for synchronously resolving resonance characteristics and device-configuration evolution across a broader range of NEMS architectures. It is also a potential tool to investigate the interfacial slipping relaxation dynamic in situ, which remains a difficult task to characterize directly during resonance measurements.

## Data Availability

All data needed to support the conclusions in the paper are presented in the manuscript. Additional data related to this paper are available from the corresponding author upon reasonable request.

## Supplementary Materials

The following supporting information can be downloaded at: https://www.mdpi.com/article/10.3390/nano16171113/s1. References [37,38,39,40,41,42,43] are cited in Supplementary Materials.
