# Supplementary material for: Synchronous In Situ Harmonic Calibration of Vibration Amplitude and Gap in 2D Nanomechanical Resonators
Source: Nanomaterials (Basel). 2026 Sep 3;16(17):1113. doi: 10.3390/nano16171113 (PMC13567318; doi:10.3390/nano16171113)
Supplement: Supplementary file 1 [file nanomaterials-16-01113-s001.zip › nanomaterials-4476994-supplementary.pdf]

Supplementary Materials for

Synchronous In Situ Harmonic Calibration of Vibration Amplitude and Gap in 2D Nanomechanical Resonators

S.I. Details of Device Fabrication and the Measurement Setup

Material preparation

The two-dimensional crystals used in this study, including hexagonal boron nitride (h-BN) and high-conductivity graphite, were grown by chemical vapor transport and purchased from Anwei Technology Co., Ltd. (Shanghai, China). Multilayer h-BN and graphene flakes were mechanically exfoliated with blue Nitto tape (Nitto Denko, SPV 224P) onto Si substrates coated with 285 nm of SiO_2. Optical micrographs were acquired using a Nikon ECLIPSE LV150NL microscope. Flat, uniform flakes were identified from their optical contrast and red, green, and blue intensity profiles extracted with NIS-Elements (Nikon) [1-3].

Fabrication of suspended h-BN/Gra heterostructure resonators

Electrode patterns were defined by electron-beam lithography (Raith eLINE Plus), followed by electron-beam evaporation of a 5/50 nm Cr/Au stack. A second electron-beam lithography step defined a 3 μm wide rectangular etching window in the SiO_2 region between the electrodes. Inductively coupled plasma dry etching then formed a 260 nm deep trench, leaving approximately 40 nm of SiO_2 at the trench bottom. Finally, the h-BN/Gra heterostructure was assembled and released over the trench using our modified polycarbonate/polydimethylsiloxane-assisted pick-up transfer process [4,5]. Heterostructure stacking and suspended-resonator assembly were completed in the same transfer workflow.

Measurement setup

Optical transduction used a 632.8 nm He-Ne laser (LASOS LGK 7634). The beam passed through an antireflection-coated viewport and was focused onto the suspended heterostructure through a 50× long-working-distance objective outside the vacuum chamber (Mitutoyo M Plan Apo SL 50×). Reflected intensity was detected with a variable-gain silicon avalanche photodetector (LBTEK PD-SAF-400A). The signal was sent to a digital lock-in amplifier (Zurich Instruments HF2LI) for synchronous acquisition of the 1ω, 2ω, and 3ω demodulator outputs. Measurements were performed at room temperature and a chamber pressure of approximately 5×10^{-3} mbar.

S.II. Branch Structure of the Effective-Gap Inversion

The complete mathematical formulation is presented in Section 2.3 of the main manuscript. In signed mathematical form, the second-to-first harmonic-intensity ratio is

$$\frac{I_{2\omega}}{I_{1\omega}} = -\cot(\gamma g)\frac{J_2(z)}{J_1(z)} \tag{S1}$$

When harmonics are measured with a lock-in amplifier, the reported intensities are the magnitudes of the signed Fourier coefficients, namely $|I_{1\omega}|$ and $|I_{2\omega}|$. The measured harmonic-intensity ratio is therefore

$$r_{21} = \frac{|I_{2\omega}|}{|I_{1\omega}|} = \left|\frac{I_{2\omega}}{I_{1\omega}}\right| = \begin{cases} -\cot(\gamma g)\frac{J_2(z)}{J_1(z)}, & \frac{I_{2\omega}}{I_{1\omega}} \geq 0 \\ \cot(\gamma g)\frac{J_2(z)}{J_1(z)}, & \frac{I_{2\omega}}{I_{1\omega}} < 0 \end{cases} \tag{S2}$$

For vibration amplitudes up to several tens of nanometers, as relevant to 2D NEMS resonators, $J_2(z)/J_1(z)$ remains positive. The function $-\cot(\gamma g)$ is periodic in g, with the spatial period

$$L \triangleq \gamma^{-1}\pi = \lambda/4 = 158.2\, nm \tag{S3}$$

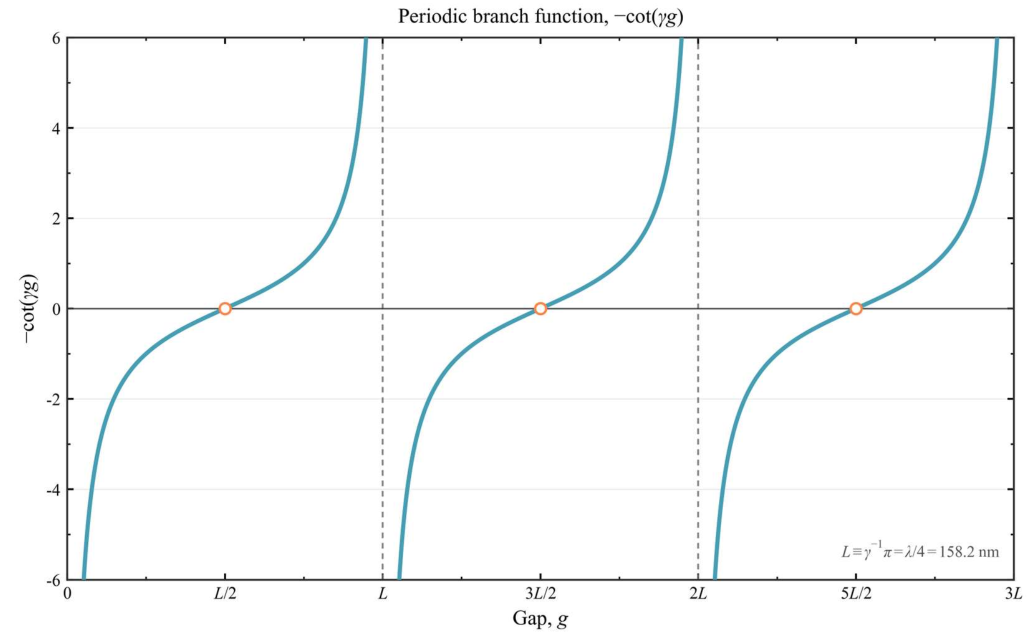

Figure S1. Dependence of $-\cot(\gamma g)$ on the gap g. The horizontal axis is marked in units of the spatial period L.

$$r_{21} = \left|\frac{I_{2\omega}}{I_{1\omega}}\right| = \begin{cases} \frac{I_{2\omega}}{I_{1\omega}}, & g \in \left[nL - \frac{L}{2}, nL\right) \\ -\frac{I_{2\omega}}{I_{1\omega}}, & g \in \left(nL, nL + \frac{L}{2}\right) \end{cases}, n \in \mathbb{Z} \tag{S4}$$

Within each spatial period L of g, corresponding to one arctangent branch indexed by n, two subbranches arise. The measured ratio is compatible with either signed mathematical ratio, $r_{21} = +I_{2\omega}/I_{1\omega}$ or $-I_{2\omega}/I_{1\omega}$.

Combining Eq. (8) of the main manuscript with Eq. (S4) gives the complete inversion expression, Eq. (S5), for the effective gap g_{eff} from the measured harmonic-intensity ratio r_{21}:

$$
g_{\mathrm{eff}} = nL + \begin{cases} \dfrac{1}{\gamma}\tan^{-1}\left(\dfrac{12\gamma x - \gamma^3 x^3}{(6\gamma^2 x^2 - 48) r_{21}}\right), & g_{\mathrm{eff}} \in \left[nL - \dfrac{L}{2}, nL\right) \\ \dfrac{1}{\gamma}\tan^{-1}\left(\dfrac{12\gamma x - \gamma^3 x^3}{(6\gamma^2 x^2 - 48)(-r_{21})}\right), & g_{\mathrm{eff}} \in \left(nL, nL + \dfrac{L}{2}\right) \end{cases}, n \in \mathbb{Z} \tag{S5}
$$

Thus, the arctangent branch index identifies a spatial period of length L, whereas the sign of the underlying ratio selects one of two half-period subbranches.

S.III. Worked Example of Vibration-Amplitude and Gap Calibration

We use a representative measured data point to illustrate the complete calibration procedure.

Equation (9) of the main manuscript is continuous and monotonic over the vibration-amplitude range of several tens of nanometers relevant to 2D NEMS resonators. Substituting the measured ratio $r_{31} = 0.00105494$ therefore gives a unique amplitude.

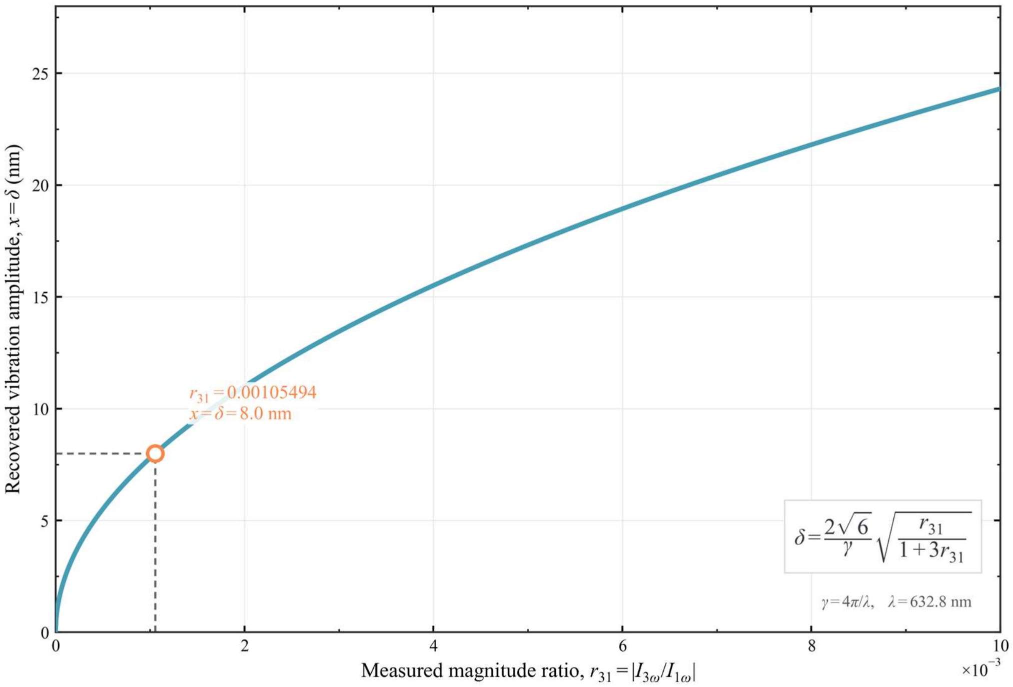

Figure S2. Schematic of the amplitude calibration principle.

Substitution into Eq. (9) of the main manuscript gives a vibration amplitude of δ = 8.0 nm.

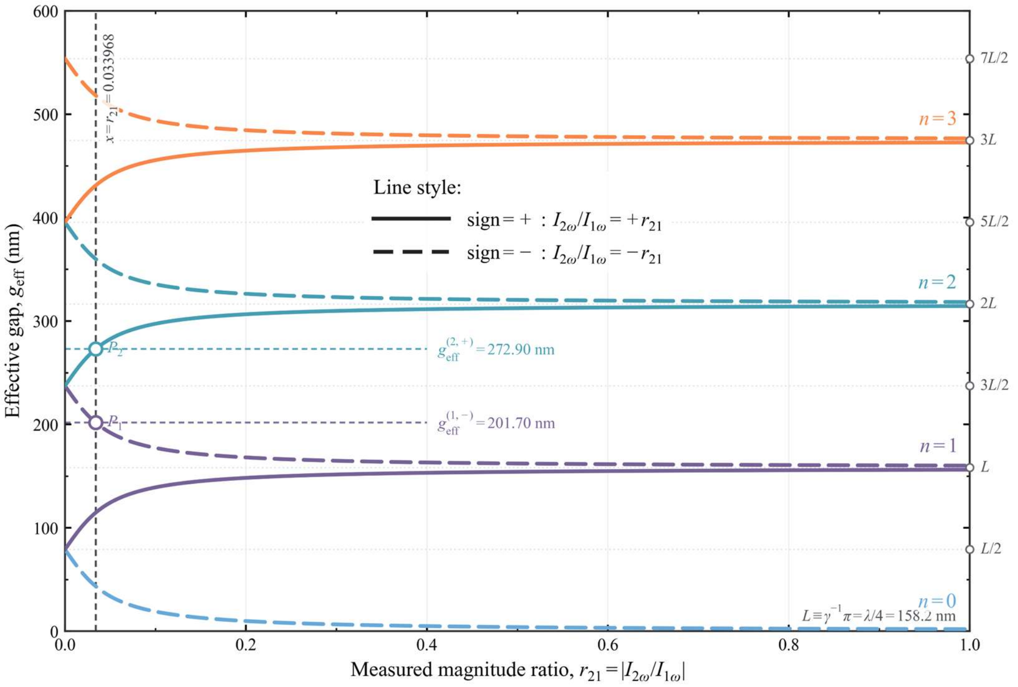

Figure S3. Schematic of retrieving the effect suspended gap from the harmonic intensity ratio via Eq. (11). Different branches indexed by n are distinguished by colors; sub-branches arising from $r_{21} = \pm I_{2\omega}/I_{1\omega}$ are distinguished by line styles. Note that the retrieval yields the effect gap g_{eff}, and when mapping it to the physical actual gap g in Fig. 3a of the main text, the offset induced by the local phase must be eliminated.

Next, substituting $\delta = 8.0\,nm$ and $r_{21} = 0.033968$ into Eq. (11) of the main manuscript yields several candidate effective gaps associated with different branches. Given the nominal trench depth of $260\,nm$, only the $n = 2, I_{2\omega}/I_{1\omega} = +r_{21}$ and $n = 1, I_{2\omega}/I_{1\omega} = -r_{21}$ subbranches are compatible with the device geometry. These subbranches give effective-gap candidates of 272.9033 nm and 201.6967 nm, respectively. They are distinguished experimentally by varying the gate voltage because stronger electrostatic attraction deflects the membrane downward and reduces the local static gap g.

The measured gate-voltage dependence selects the n = 2, $+r_{21}$ subbranch, giving an effective gap of 272.9033 nm.

Finally, applying the branch-local phase correction $\varphi_2/\gamma = 54.7195\,nm$, gives the local static gap $g = g_{eff} - \varphi_2/\gamma = 218.1838\,nm$.

S.IV. Parameters Used in the Multilayer Thin-Film Interference Model

Table S1. Structural parameters and complex refractive indices used in the multilayer thin-film interference model.

**Layer number (incident side first)**	**Material**	**Thickness**	**Complex refractive index at $\lambda = 632.8\ nm$**
1	Vacuum	Semi-infinite	$1 + 0i$
2	h-BN	$3.40\ nm$ (~10 layers)	$2.13 + 0i$ [6,7]
3	Graphene	$1.02\ nm$ (~3 layers)	$2.73 + 1.356i$ [8]
4	Vacuum gap	$\mathrm{g} + \delta \sin(\omega t)$	$1 + 0i$
5	SiO_2	$40\ nm$	$1.46 + 0i$ [3]
6	Si	Semi-infinite	$3.87 + 0.016i$ [9]

S.V. Sensitivity of the Inversion to Residual SiO_2 Thickness

We further examined the sensitivity of the branch-local correction phase φ_n and hence the recovered local static gap g to the residual oxide thickness d_{ox}.

In the multilayer thin-film interference model, the SiO_2/Si composite substrate forms the lower reflecting interface. Its effective amplitude reflection coefficient is ${r_{4,5}}^{\mathrm{eff}} = \frac{r_{4,5}+r_{5,6}\exp(2i\beta_5)}{1+r_{4,5}r_{5,6}\exp(2i\beta_5)}$. Here, ${r_{4,5}}^{\mathrm{eff}}$ denotes the effective coefficient of the complete SiO_2/Si structure viewed from vacuum. The terms $r_{4,5}$ and $r_{5,6}$ are the vacuum-SiO_2 and SiO_2-Si interface coefficients, respectively. The quantity β_5 is the one-way phase delay acquired in layer 5, the SiO_2 layer. Figure S4 shows how the resulting intensity reflectance and reflection-coefficient phase depend on residual oxide thickness d_{ox}.

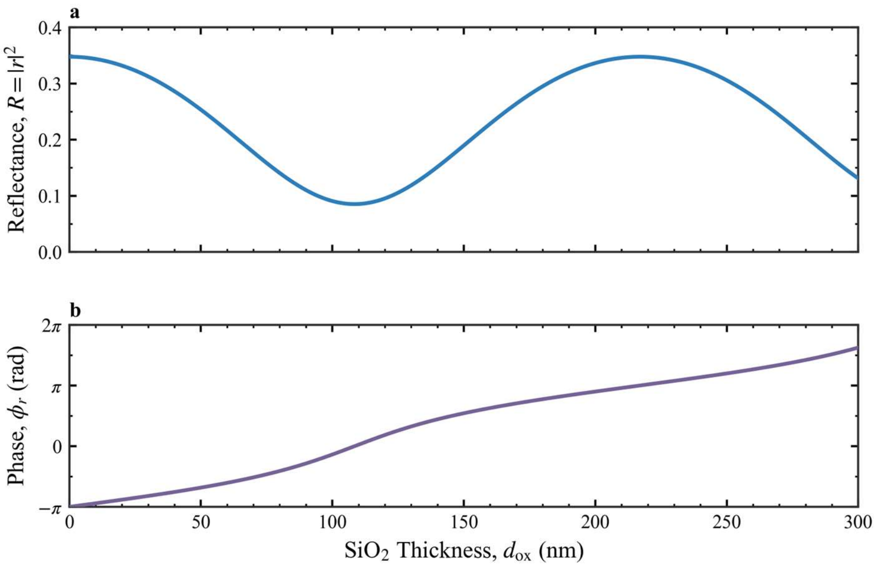

Figure S4. Intensity reflectance (a) and phase of the amplitude reflection coefficient (b) of the SiO_2/Si composite structure for 632.8 nm light incident from vacuum.

Physically, the multilayer thin-film interference model describes interference between the h-BN/Gra heterostructure and the lower SiO_2/Si interface. The residual oxide thickness d_{ox} primarily changes the phase of the effective amplitude reflection coefficient of the lower composite interface. This phase change is captured by the branch-local phase correction.

We calculated the branch-local correction phases for the $n = 1, I_{2\omega}/I_{1\omega} = -r_{21}$ and $n = 2, I_{2\omega}/I_{1\omega} = +r_{21}$ subbranches over a range of residual SiO_2 thicknesses [Fig. S5(a)]. Their corresponding effective-gap offsets are shown in Fig. S5(b).

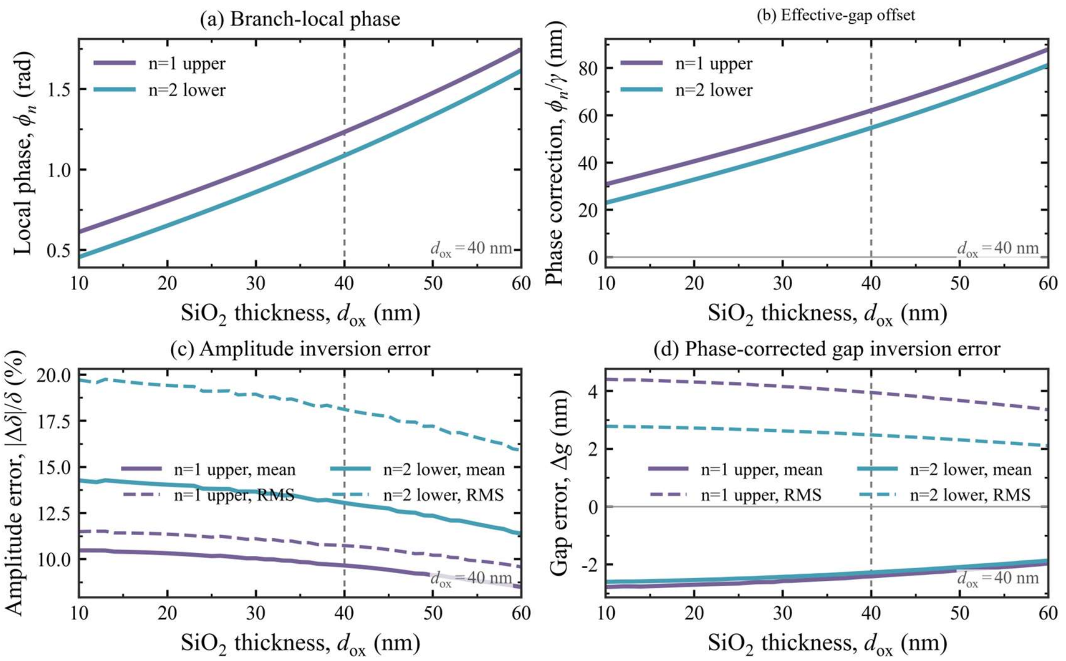

Figure S5. Effect of residual SiO_2 thickness d_{ox} on (a) branch-local correction phase φ_n, (b) effective-gap offset, (c) amplitude-inversion error, and (d) phase-corrected gap-inversion error.

We also quantified the amplitude and phase-corrected gap errors induced by d_{ox} when applying the effective two-beam inversion [Fig. S5(c,d)]. Across the examined thickness range, both error metrics vary smoothly and remain of the same order as those obtained at the nominal device thickness.

References

1. M. Mondal, A. K. Dash, and A. Singh, ACS Nano 16, 14456 (2022).
2. R. V. Gorbachev, I. Riaz, R. R. Nair, R. Jalil, L. Britnell, B. D. Belle, E. W. Hill, K. S. Novoselov, K. Watanabe, T. Taniguchi, A. K. Geim, and P. Blake, Small 7, 465 (2011).
3. P. Blake, E. W. Hill, A. H. Castro Neto, K. S. Novoselov, D. Jiang, R. Yang, T. J. Booth, and A. K. Geim, Applied Physics Letters 91, 063124 (2007).
4. Y. Zhang, Q. Liu, Y. Xiao, J. Fang, Z. Shang, Z. Guo, F. Hu, Y. Liu, G. Deng, M. Zhu, Z. Zhu, X. Wang, F. Luo, and S. Qin, Applied Physics Letters 126, 223505 (2025).
5. Q. Liu, W. Xu, X. Li, T. Zhang, C. Qin, F. Luo, Z. Zhu, S. Qin, M. Zhu, and K. S. Novoselov, International Journal of Extreme Manufacturing 6, 015501 (2024).
6. P. G. Zotev, Y. Wang, D. Andres-Penares, T. Severs-Millard, S. Randerson, X. Hu, L. Sortino, C. Louca, M. Brotons-Gisbert, T. Huq, S. Vezzoli, R. Sapienza, T. F. Krauss, B. D. Gerardot, and A. I. Tartakovskii, Laser & Photonics Reviews 17, 2200957 (2023).
7. Y. Rah, Y. Jin, S. Kim, and K. Yu, Optics Letters 44, 3797 (2019).
8. J. W. Weber, V. E. Calado, and M. C. M. van de Sanden, Applied Physics Letters 97 (2010).

9. M. A. Green, Solar Energy Materials and Solar Cells 92, 1305 (2008).
